# Macrolepidoptera biodiversity in Wooster, Ohio from 2001 through 2009

**DOI:** 10.3897/zookeys.452.8009

**Published:** 2014-11-05

**Authors:** Roger A. Downer, Timothy A. Ebert

**Affiliations:** 1 Department of Entomology, The Ohio State University, 1680 Madison Ave, Wooster, OH 44691; 2 Current address: Department of Entomology, University of Florida, 700 Experiment Station Rd., Lake Alfred, FL 33850

**Keywords:** Organismal biological diversity, survey, seasonality, phenology, moth

## Abstract

A Skinner mercury vapor light trap was operated from 2001 through 2009 in a residential backyard to document biodiversity within the moth families Thyatiridae, Drepanidae, Geometridae, Mimallonidae, Apatelodidae, Lasiocampidae, Saturniidae, Sphingidae, Erebidae (including Lymantriinae and Arctiinae), Euteliidae, Nolidae, and Noctuidae. When making comparisons to older literature, we recalculated our results to conform to the older classification of the Noctuoidea. Moths were released after identification. There were 501 species documented in 77581 captures from 1290 sampling dates. There was a perceived risk that released moths would fly back into the trap the following evening. This should result in an abnormal number of rare moths that are caught multiple times. The number of species caught twice versus the number caught once was no different than a similar ratio for surveys that used more traditional sampling methods. Therefore this concern does not seem to be valid for these data. These data are provided in a supplementary file available for download.

There were three previous surveys conducted in nearby natural areas. They documented fewer species than were documented here. To understand this better, we examined several specialized groups of moths that tend to use host plants not typically found in an urban residential yard. More species in *Schinia* Hübner, *Catocala* Schrank, *Acronicta* Ochsenheimer, and Herminiinae Leech were found in this survey than the other local surveys. Only in the *Papaipema* Smith did we recover fewer species, though it was still above 70% of what was expected. This diversity could be a result of sampling effort, but it shows that this urban location has a very diverse moth fauna. We suggest that this diversity is partly due to the planting of native plant species in the area about the light trap. Therefore we would concur with others that urban landscapes can be planned to increase biodiversity relevant to more natural ecosystems.

In this study we looked at the ratio of the number of species of Geometridae divided by the number of species of Noctuidae as one approach to evaluating the level of disturbance in the moth assemblage. Although the yearly average was nearly constant, the seasonal ratio ranged from 0.09 to 0.91 depending on the sampling date. We also calculated alpha diversity and found that seasonal change in alpha diversity greatly exceeded yearly differences. This strong seasonal component means that a comparison between two studies requires a correction for seasonality and similar sampling intervals. In this study, a shift of two weeks would be sufficient to result in a significant difference in alpha diversity. This is the equivalent of increasing temperature by 1.53 °C. Seasonal shifts limit the usefulness of this methodology for environmental assessment because the within season change exceeds the between season change. This problem is compounded when sampling designs interact with this seasonality.

In describing our data, we made use of a growing degree day (GDD) model. This approach corrects for simple temperature dependent shifts in moth biology. Consequently, some of the variability in the data was removed, which should improve the power of statistical tests involving survey data. If sampling protocols were based on growing degree days rather than calendar dates, the bias caused by temperature induced shifts in seasonal cycles could be reduced.

## Introduction

Moths play an important role in ecosystems. Adults pollinate flowers, and their larvae play a variety of roles as herbivores, detritivores, omnivores, or carnivores (Triplehorn and Johnson 2005). Moths are an important food resource for a variety of animals including lizards, small mammals (Kronfeld-Schor and Dayan 1999), birds (Schwenk et al. 2010; Visser et al. 2006), bats (Dodd 2006; Dodd and Lacki 2007; Dodd et al. 2008), and other insects (Howell and Davis 1972). Because of their pivotal role in ecosystem function, moths are sometimes used for assessing the effects of environmental change (Gimesi et al. 2012), habitat restoration (Highland and Jones 2014), or environmental impact assessment (Chaundy-Smart et al. 2012; Kitching et al. 2000; Slade et al. 2013).

The largest family of moths is the Noctuidae (Borror et al. 1976). However, the classification of the Noctuidae and closely related families has been extensively revised in recent years (Lafontaine and Schmidt 2010). Such revision improves our understanding of the biology of this diverse group of moths, and we will use the new classification when describing our results when there are no comparisons to older literature. However, we will use the older classification for the Noctuoidea when comparing our results to the older literature. If we cite a manuscript we will use the classification scheme that was used in the cited article. The old families Lymantriidae and Arctiidae are now two subfamilies of the Erebidae, and the old family Noctuidae now consists of the families Erebidae, Euteliidae, Nolidae, and Noctuidae (Lafontaine and Schmidt 2010).

Urbanization results in a large number of environmental changes. Physical changes from urbanization include elevated pollution levels in air and soil, elevated temperatures, increased soil compaction, and increased soil alkalinity (McKinney 2002). Biotic changes include biological deserts (roads, parking lots, and buildings), loss of native host plants, reduction in patch sizes of suitable habitat, and the introduction of weedy species and ornamentals (McKinney 2002). This might result in a taxonomic homogenization through loss of specialists and an over-representation of generalists (Marie-Hélène et al. 2011). Thus urbanization is a biotic filter that favors a few generalists and excludes many species adapted to specific native environments (McKinney 2006; Niell et al. 2007). However, a decrease in biodiversity with increasing urbanization is not always observed (Krauss et al. 2003). Furthermore, sometimes elevated biodiversity is observed somewhere between the natural areas at the periphery of human habitation and the urban core. One explanation for this is the intermediate disturbance hypothesis where human disturbance creates more biotic boundaries and increased environmental heterogeneity. It is also possible to have greater diversity at the urban core relative to closely adjoining areas because new development in the adjoining areas tends to remove most of the existing vegetation, increases soil compaction, and removes topsoil (McKinney 2002). Such unnatural increases in biodiversity can be misleading when discussing biodiversity loss due to urbanization. Urbanization destroys key habitats that harbor specialists, and a simple count of the number of species may obscure loss of native biodiversity if an urban area is invaded by a diverse assemblage of generalists that can better utilize the exotic vegetation (McKinney 2006). These ideas have been tested through habitat manipulation. Replacing non-native vegetation with native species can quadruple insect abundance and triple biodiversity (Burghardt et al. 2008). Improved biodiversity in urban settings from habitat manipulation that favored native species was also observed in Finland (Valtonen et al. 2007). Thus, although urbanization can result in biodiversity losses, even small plantings of native species within an urban setting can mitigate these effects in localized areas (Tallamy and Shropshire 2009).

Biodiversity is one measure of the effect of environmental impact, but it can be distorted by an influx of new generalist species better adapted to disturbed environments. It has been suggested that the ratio of the number of geometrid moths to the number of noctuid moths is a better measure of environmental disturbance (Kitching et al. 2000). The idea was that noctuids tend to be larger moths capable of greater dispersal and they generally have a broader host range than the geometrids. The influence of body size on dispersal was examined quantitatively by measuring moth migration between small islands (Nieminen 1996), but dispersal ability does not always equate to migration rates (Slade et al. 2013). Kitching showed that uncleared remnants had a Geometrid:Noctuid ratio of 0.987, cleared remnants 0.682, and scramberland remnants 0.186 (scramberland sites are covered by *Lantana camara* L., *Solanum mauritianum* Scop., and a variety of other weeds with a high proportion of exotic species. Isolated rain forest shrubs and trees emerged from this understory). This ratio was proposed as a first approximation, and a more restricted list of moths in specific subfamilies within the Geometridae and Noctuidae were detailed as a more refined approach. Others have proposed similar indicators, though typically selecting specific groups within these and other families (Summerville et al. 2004).

Moth surveys are often justified as tools to document ecological processes like climate change (Fox et al. 2011), environmental impacts (Summerville 2011; Taki et al. 2010), and habitat restoration (Bucheli et al. 2006; Summerville 2008). In trying to integrate our results with these other studies, there are well-known problems associated with trapping methodology: type of light trap (Fayle et al. 2007; Leinonen et al. 1998; von Langevelde et al. 2011), number of trapping nights, number of traps, and environmental factors like moon phase (Sanyal et al. 2013), or artificial lighting (Schweitzer et al. 2011). However, seasonal variability, or more precisely incomplete seasonal coverage in most surveys, can result in major systemic errors (Summerville 2008), and this effect makes study-to-study comparisons problematic.

To put this survey in perspective, we compiled a table of several moth surveys from the last 70 years (Table 1). These surveys were from a wide variety of habitats, and not all collections were restricted to black light trapping of macrolepidoptera. The ratio of number of species of Geometridae divided by Noctuidae was very consistent with a ratio of 0.46 and a standard deviation of 0.14. The extreme values were from the Maine survey that was 2.5 standard deviations below this value whereas the West Virginia survey from Camp Dawson Collective Training Area was 1.9 standard deviations above.

**Table 1.** Overview of moth surveys including number of moths sampled (no.), number of species recorded (spp.), and the number of species of Noctuidae (Noct.) and Geometridae (Geo.). The main focus was surveys from the United States.

Cite	State	Location	No.	Spp.^1^	Noct.	Geo.
A	OR	Blue Mtns	20322	383	212	93
B	WV	Cooper’s Rock State Forest	29983	400	220	102
C	WV	Turkey Run and Great Falls National Pks^2^	Unk	480	278^3^	107
C1	WV	Camp Dawson Collective Training Area	3666	235	101	73
C2	WV	Southern West Virginia^2^	Unk	751	418	191
D	FL	Blue Spring State Park^2^	Unk	275	171	67
E	NJ	Hutcheson Memorial Forest	22880	410	253	98
F	LA	West Feliciana Parish	3155	314	122	68
G	LA	Long-leaf pine Savanna	1182	208	84	42
H	IN	Morgan-Monroe State Forest	14537	324	110	72
I	IA	Neal Smith National Wildlife Refuge	9416	508	136	69
J	OH	Wilderness Center^2^	Unk	413	233	94
K	OH	Funk Bottoms^2^	Unk	262	159	46
L	OH	Atwood Lake State Park^2^	Unk	376	221	93
--	OH	Wooster (current study)	77581	501	314^4^	104
M	TN,NC	Great Smoky Mountains National Park^2^	Unk	914	528	225
N	AR	Ozark mtns	8720	314	57^5^	33^5^
O	Hungry	Aggtelek National Park	127035	994	512	326
P	Canada	Ministik Hills, Alberta	24578	264	151	66
Q	Canada	Acadia Research Forest, New Brunswick	31634	539	270	169
R	ME	Orono	43435	337	258	27

Citations: A (Grimble et al. 1992) B (Butler and Kondo 1991) C (Steury et al. 2007) C1 (Butler et al. 2002) C2 (Albu and Metzler 2004) D (Profant 1989) E (Moulding and Madenjian 1979) F (Landau and Prowell 1999b) G (Landau and Prowell 1999a) H (Summerville et al. 2008) I (Lewis et al. 2005) J (Rings et al. 1987) K (Williams et al. 1977) L (Rings and Metzler 1988) M (Scholtens and Wagner 2007) N (Dodd et al. 2008) O (Szabo et al. 2007) P (Schmidt and Roland 2006) Q (Thomas 2001) R (Dirks 1937)

1 The published species counts often included families that were not part of this research. Therefore the number of species were recounted and species from families not part of this study were removed.

2 Survey only, no abundance data presented.

3 Nolidae was separated in this list, and these were added back into the Noctuidae to get this number.

4 using older classification (Hodges 1983). Revised values for Noctuidae are 208, giving a ratio of 0.5.

5 These are minimums, some material not identified to species.

There have been three Lepidoptera surveys in our local area. These took place at Funk Bottoms, The Wilderness Center, and Atwood Lake Park. Funk Bottoms Wildlife Area consists of periodically flooded moist meadows, bottomland hardwoods, and 80 ha of permanent marsh. However, thousands of hectares may be flooded for up to several months each year (Williams et al. 1977). This site was about 13 km SW of our light trap. Black light trapping was done at two locations from April through November in 1995 for a total of 30 trapping nights. The Wilderness Center features about 40.5 ha of virgin forest and a stream. Management programs have created a pond, a lake, and about 2 ha of thicket (Rings et al. 1987). The Wilderness center is about 25 km SE of our light trap. Collecting was done from 1977 through 1985. In 1984 and 1985, trapping was done at five sites by black light trap and sugaring. Light traps were run twice per week from May through October 1984 (24 sample nights), and March through June 1985 (16 sample nights). Atwood Lake Reservoir was constructed in 1937 on Indian Fork Creek. It had a natural oak-hickory and beech-maple woodlands that underwent a reforestation effort using pine and *Liriodendron tulipifera* L. (Rings and Metzler 1988). The Wildlife Area Atwood Lake Park was about 58 km SE of our light trap. Trapping was done at four locations by black light and sugaring on no more than 21 nights in 1985 and 14 nights in 1986. The primary repository of specimens from the study at the Wilderness Center was the Wilderness Center collection. Additional specimens were deposited in the reference collection at the Ohio Agricultural Research and Development Center (OARDC) (1680 Madison Ave, Wooster, Ohio). Specimens from the other studies were deposited in the OARDC reference collection. Subsequently, many of the OARDC specimens were relocated to the Museum of Biological Diversity at The Ohio State University.

Temperature plays a critical role in biological processes. A “growing degree day” (GDD) model is typically used where accumulated thermal units are explanatory variables for the biological process of interest (Forrest and Thomson 2011; Harrell et al. 2011; Kimball et al. 2012; Smitchger et al. 2012; Spear-O’Mara and Allen 2007). Therefore we used a growing degree day model to change calendar date into a variable more relevant to insect biology and examined biodiversity in this light. Using a growing degree day approach also allowed a more natural grouping of multi-year data because it corrects for yearly shifts in accumulated heat units (Kimball et al. 2012). Therefore we would expect that a GDD approach would result in less variability in the data relative to the mean response. This should improve the sensitivity of statistical tests in a variety of applications that use biodiversity estimates to assess environmental conditions. This approach can also distinguish between thermally induced shifts in life cycles versus a disruption of those life cycles.

It would be useful to define the area being sampled when conducting any sampling activity. Defining the sampling radius about a light trap is not simple in part because it is a probability function where the probability of capture decreases exponentially with increasing distance. The probability of capture also declines rapidly if the moth starts its movement outside the radius where the light is strong enough to be attractive. Anything that affects background light levels (moon phase, light pollution, cloud cover) will alter capture probabilities (Steinbauer et al. 2012; von Langevelde et al. 2011; Yela and Holyoak 1997). Estimates of attraction radii range from 3m to 800 m. Attraction radii are also species specific (Baker and Sadovy 1978; Beck and Linsenmair 2006; Truxa and Fiedler 2012). In recapture experiments, less than half of the moths released 5 m or less from the light were recaptured, and less than 20% were recaptured at 25 m (Truxa and Fiedler 2012). Other studies have estimated attraction radii of between 200 m at full moon to 520 m at no moon (Bowden 1982). Exact distances vary by trap type (wavelength, power, design), trap height, species, and environmental factors influencing the contrast between ambient light and trap light (Fayle et al. 2007; Hollingsworth and Hartstack 1972; von Langevelde et al. 2011; Yela and Holyoak 1997).

The above paragraph contains considerable uncertainty about the exact attraction radius. This is caused by differences in the methodology of the cited works. We provide two cases to illustrate the point. Baker and Sadovy (1978) used a 125W mercury vapor lamp using mark-recapture methods, and 5000 individuals of *Noctua pronuba* (L.) and *Agrotis exclamationis* (L.). Multiple traps were placed about a release point using two configurations. A sharp decline in the number of recaptures was observed starting at 5 meters if the light traps were dispersed about the release point. The other approach used two light traps, one closer to the release point than the other. In this case the further light trap ceased to capture any moths if it was more than 7 meters from the release point. In contrast, Truxa and Fielder (2012) used a mark recapture method, but traps with two 15W black lights were used. They used these traps to capture 2331 moths from 166 different species for subsequent marking and releasing. Two experiments were run, the first in a deciduous tree forest at University of Bayreuth with tree heights from 5 to 8 m. Moths were trapped, identified, marked and released 24h after capture. A single light trap was placed along a gravel path and moths were released at 13 distances from 2 to 40 m distant. The second experiment was done in a deciduous tree forest at the Donau-Auen National park along a straight forest road. The same type of trap was used, but there were 12 release points from 5 to 100 m distant. In the first experiment 20% of the moths released at 35 m were recaptured, but none of the moths released at 40 m were recovered. In the second experiment, no moths released past 80 m were ever recovered. Baker and Sadovy used two species but moths were allowed to go in any direction. Truxa and Fielder used many species but the cleared forest path forms a tunnel that could funnel moths towards the trap. None of the cited experiments are flawless, but they all indicate that the attraction radii of most traps will be fairly limited. Elevated traps may have larger attraction radii (Baker and Sadovy 1978), but the attraction radii of elevated traps is not relevant to this study. From another perspective, anyone who has held and released a moth will point out that many of these moths have the ability to fly much further than a few hundred meters. However, that is not the point. This is about the probability of capturing a moth that starts its flight activity some distance from the light. That probability declines rapidly with increasing distance. The cited studies suggest that the probability is very low past a few hundred meters.

Our goals were to; 1) Document biodiversity in an urban setting to compare to three previous surveys in natural settings. 2) A quantification of the effect of seasonal changes in moth diversity. 3) Document the utility of a phenological model in understanding biological survey results.

## Materials and methods

The trap was located in an urban (as defined by US Census Bureau (Anonymous 2010)) setting in Wooster, Ohio, USA (40.80917°N by 81.93722°W), population 26,000 (www.city-data.com viewed 21/7/2010). The residential back yard was 0.16 ha of lawn on the Killbuck-glaciated Pittsburgh Plateau (http://www.dnr.state.oh.us/portals/10/pdf/physio.pdf) at 353 m elevation (http://www.usgs.gov). The acreage was determined using the Wayne County Auditor’s assessment of lot size less the auditor’s measure of the size of the house (Waynecountyauditor.org viewed Nov 2010). Neighboring parcels were smaller than this one with an average parcel size (including the house) of 0.127 ha (standard deviation 0.059). The neighborhood contained mature trees and shrubs including oaks, ash, locust, cherry, conifers, maples, blueberries, lilacs, and dogwoods. Much of the neighborhood was dominated by turf grass and associated weeds (Cheng et al. 2008). The yard with the trap had a variety of native and non-native annuals and perennials, and a small (about 2 meter diameter) artificial pond/marsh area. The garden was developed gradually beginning in 1993, and one goal in selecting plants for this garden was to provide nectar and larval food resources for a variety of native pollinator species. Such activities are known to increase biodiversity in urban landscapes even on small 0.13 ha parcels (Burghardt et al. 2008), though the biodiversity benefit of specific activities can sometimes be variable (Gaston et al. 2005) despite a general observation that plant biodiversity increases insect biodiversity in natural habitats (Schaffers et al. 2008).

Moths were collected using a Skinner mercury vapor light trap with a 125 Watt mercury vapor bulb (model 7591 from Watkins and Doncaster (www.watdon.co.uk)) with the filament 33 cm above ground level. The performance of this trap relative to others was recently evaluated (Fayle et al. 2007). The trap was run most nights when temperatures were above freezing and there was no rain. Moths were identified and most were released on the other side of the house on the morning after the trap was run, about 20 m distant. There were street lights on the eastern side of the house where moths were released. Voucher specimens for the new county records were retained and deposited with the Museum of Biological Diversity, The Ohio State University, 1315 Kinnear Rd. Columbus, OH, USA 43212. These records were additions to earlier work on the moths of Ohio (Rings and Downer 2001) see http://www.oardc.ohio-state.edu/rb1192/default.asp (accessed 6, September 2009). Additional vouchered records for most Ohio Noctuidae and Erebidae were published earlier (Rings et al. 1992). Although physical specimens don’t exist for the remaining identifications, photographic documentation for many specimens can be found at www.butterfliesandmoths.org. Below the banner click on regional checklists. Then select the region United States/Ohio/Wayne, click apply. From the checklist for Wayne County, click on the species of interest and proceed to another page. Scroll down and click on Sightings Table where all the sightings for the species are listed. Scroll through these to find the records for submitter “rogerdowner”.

We suggest using the GPS coordinates provided earlier and Google Earth® (http://www.google.com/earth/index.html) for a detailed view of the environment about the moth trap. Botanical composition of nearby parks (1 km distant) is largely irrelevant due to the short attraction radii of black light trapping methods (<520 m). Furthermore, the light was close to the ground, so buildings, trees, and tall shrubs all block light and serve to further restrict this radius.

Moths were identified and catalogued using an older classification system (Hodges 1983), that was subsequently updated (Lafontaine and Schmidt 2010). In a few cases this required personal communication with Dr. Lafontaine to correct our species list. The older system was retained when making comparisons to the older literature. In this system Arctiidae and Lymantriidae are separate families. New results utilize the newer classification where the Arctiidae and Lymantriidae become subfamilies in the Erebidae, and the old Noctuidae is divided into the Erebidae, Euteliidae, Nolidae, and Noctuidae.

### Phenology

A lower developmental threshold of 10 °C was used to estimate growing degree days (GDD) (Pruess 1983). Weather data were measured at a weather station located at the OARDC about 8 kilometers south of the trapping site. The method used to calculate GDD was a modified sign wave method (Allen 1976; Pruess 1983) as outlined at http://www.oardc.ohio-state.edu/gdd/glossary.htm (viewed Jan 2009) and see also (Cardina et al. 2007). We recognize that many of the moths may have developmental thresholds different from 10 °C, but for consistency, we retain the base temperature of 10 °C even for those few species where sufficient research exists to justify a different base. The calculation for GDD in the OARDC site was based on English units, which were converted to metric using GDD in °C = -0.00013+0.555639* GDD in °F (*F*=57017608; df 1,363; *P*<0.0001). We used the single triangulation method in cases where we needed to recalculate GDD (Lindsey and Newman 1956), and note that there tends to be close agreement between the various sine and triangulation methods (Roltsch et al. 1999). The use of a fixed threshold temperature for different species has been used to model voltinism changes in Finnish moth species (Poyry et al. 2011).

### Analysis

We used the various approaches to estimating species richness implemented in EstimateS (Colwell 2013) set to run 1000 randomizations without replacement. We calculated species richness using both individual sampling dates and yearly pooled samples. However the difference between the estimates was less than the estimated standard deviation for either method. Therefore we only present results using individual sampling dates.

We used the proportion of species represented by a single capture as an indication of the effectiveness of the sampling protocol (Carlton et al. 2004; Williams et al. 2007). This approach assumes that no viable moth population can be represented by a lone individual, so the capturing of only a single individual indicates that the method missed some individuals. Although some singletons are indicative of an ineffective sampling methodology, e.g., moth species that do not come readily to light, some singletons should be expected since they could come from migrating individuals that have little interest in the trap or its environment.

The study site had bats, birds, and wasps that preyed on moths attracted to the light. There may also have been other vertebrate and invertebrate predators. Moths were released in different locations in the yard to reduce such predation. However, we could not quantify the level of predation or the effectiveness of any effort at reducing predation. Sometimes moths were too worn to be properly identified, and these individuals were ignored.

## Results and discussion

### Potential problem

We expect that three traps run six times per year for one year (Summerville and Crist 2003) would have less impact on the local ecosystem than would one trap run at the same location 115 to 215 times per year for nine years (this study). Long term intensive sampling has shown the potential to negatively impact moth populations (Vaisanen and Hublin 1983). Consequently moths were released after identification. This methodology may inflate abundance estimates, though it would not affect the number of observed species. So we address the issue of multiple captures internally using the frequency of doubletons, and externally by comparing with the published literature.

Quantitative assessment of the effect of multiple captures was made by examining the number of moth species captured once per year versus the number represented by two captures per year. A methodology that increased the probability of recapturing moths should have a disproportionate number of rare species captured twice. The average doubleton÷singleton ratio for each year was 0.574 (standard deviation [SD] of 0.211). We also look at this ratio for each sampling date because in this case doubletons cannot be recaptures of the same individual. The doubleton÷singleton ratio for each night where there were both singletons and doubletons was 0.382 (SD 0.336). The yearly average was not significantly greater than the daily average (*F*=2.91; df=1, 980; *P*=0.09). Obviously, a failure to detect a significant difference is not the same as proving that there was no effect. Summerville et al. (2003) reported a ratio of 0.568 (SD of 0.054), whereas Summerville et al. (2004) found a ratio of 0.472 (SD of 0.092) (these numbers based on data provided by Dr Summerville from research published in cited literature). Lower values have been observed in other studies, 0.311 [Vancouver, Canada] (deWaard et al. 2009), 0.552 (SD of 0.247) [Rothamsted insect survey site 336, United Kingdom] (Harrington and Woiwod 2007), as well as higher values 0.932 [Blue Mts, Oregon] (Grimble et al. 1992), 0.618 [Birch Mts., Alberta, Canada] (Macaulay and Pohl 2005). A collection from Inverness Ridge in California had a value of 0.362 (data provided courtesy of Jerry Powell). A collection from Annville, Pennsylvania had a value of 0.222 (data provided courtesy the Pennsylvania Natural Heritage Program) (Ferster et al. 2008). The most comparable study would be the 31 years of data from Rothamsted because the data were yearly counts over multiple years from one locality. We conclude that our result of 0.574 is not unusual compared to these studies, and therefore the possibility of capturing the same individual twice doesn’t seem to result in an excessive bias in this study. However, we don’t know if we got lucky, or if this is a typical result.

### Raw data

The raw data are included as supplemental data. The data file is in Excel format. We recommend that users read the “Introduction”, which is the first page (left-to-right) in the file. The next page to the right in the file includes the weather data. Farther to the right are nine pages with yearly capture data. These pages include the number of growing degree days accumulated by each collection date. Cells are blank if no individuals of a given species were captured on a specific date. Next is a page “Condensed List” that contains total number of each species, and the number of years each species was collected. This page contains the species as they were identified and the equivalent under the system by Lafontaine and Schmidt (2010). It also lists the range in capture date, and range in growing degree days. Then follows total captures per year, and a list of known host plants. Next there is a list of the 13 new county records and their collection date. Next is a page with a list of univoltine and bivoltine species selected based on abundance and environmental fidelity. Next is a listing of the 20 pest species and their yearly abundance. This was extracted from the main list to facilitate access. Lastly is a page with the moon phases. We did not find this of any use, but it may prove useful to someone else.

### Diversity and abundance

In 1290 sampling dates from 1 January 2001 through 31 December 2009, a total of 77,581 moths were captured and identified. This averages to 60 moths/night. However this number has little value because it includes early and late season samples that have few moths. In 2001 the range was from 1 to 496 moths per night with an average of 96. Within this nine year sampling effort were 501 species, of which 122 were found in all nine years.

The numbers of species within a family that were represented by a single capture has been used as a metric for evaluating the effectiveness of a sampling methodology. Carlton et al. (2004) reported that singletons accounted for 38 to 43% of their sample, and this was considered indicative of sufficient sampling effort. Using this criterion, the average singleton rate per year for the Thyatiridae, Drepanidae, Mimallonidae, Apatelodidae, and Saturniidae all indicate that the sampling strategy might be ineffective. Either the moths do not respond well to black light traps, or we may be sampling transients. The Noctuidae and Geometridae accounted for the majority of singletons (Table 2). However, relative to the number of species in these families, the number of singletons in these families was low, thereby indicating sufficient sampling effort. The Lasiocampidae, Sphingidae, Erebidae, Euteliidae, and Nolidae also had singleton percentages that were with acceptable limits. The estimated total number of species was between 533 for the Bootstrap method and 599 for the Jacknife 2 method (Table 3).

**Table 2.** Genera, species, and abundance compositions for 12 Families of macrolepidoptera in Wooster Ohio. Total percentage singletons is the number of species represented by a single capture in the nine years of the survey divided by the number of species. Average percentage singletons is the average of the number of singletons caught each year divided by the number of species caught that year.

Family	Individuals captured	Number of genera	Number of species	Total percentage singletons	Average percentage singletons
Thyatiridae	16	3	3	33	50
Drepanidae	31	2	2	0	43
Geometridae	8578	70	104	13	20
Mimallonidae	3	1	1	0	100
Apatelodidae	8	2	2	0	63
Lasiocampidae	229	3	5	0	3
Saturniidae	42	8	8	25	50
Sphingidae	184	9	13	7	41
Notodontidae	2755	18	32	16	22
Erebidae	17197	11	112	15	23
Euteliidae	112	3	5	0	34
Nolidae	340	3	6	0	21
Noctuidae	48086	122	208	11	22

**Table 3.** Diversity statistics^1^: Estimates of the number of species.

Statistic	Mean	Lower 95% CI	Upper 95% CI
Chao 1 Mean	553.69	529.61	598.78
Chao 2 Mean	560.43	533.43	609.52
		**Standard Deviation**	
Jacknife 1	568.95	8.33	
Jacknife 2	598.97	1.18	
Bootstrap	533.27	0.49	

1 (Colwell 2013)

The number of species only present in a single year was greatest in 2001 (Table 4). At the other extreme, 2005 and 2006 had an unusually small number of species that were only captured in that year. For the first six years, the number of species never before captured declined (Table 4). Eventually it should converge to the speciation rate plus the immigration rate of new species. However, 2007 and 2009 were unusual years in that more new species were added than one would expect from the initial pattern. These two years therefore have a large influence on the estimated total number of species. There was no obvious pattern in the new species for 2007 and 2009. None of the species were pests. In 2007, four of the 15 species fed on oak, maple, or walnut, whereas six of the 15 species from 2009 had these hosts. Seven of the 15 species in 2007 were captured again in either 2008 or 2009. In 2003 there was an F2 tornado that went through the city. Another F2 was within a few miles of the city in 2009. Smaller tornados occur in Wayne County nearly every year (http://www.tornadohistoryproject.com/tornado/Ohio/2003/map). Another environmental disturbance happens twice per year as the city applies insecticide for mosquito control using a truck mounted fogger. None of these events seem related to patterns in our survey.

**Table 4.** Summary by year, and over the nine year study period for macrolepidoptera in Wooster Ohio. We list the number of days sampled (Days), number of individuals captured (Captured), number of genera (Genera), number of species (Species), the species that had never been captured prior to that year (Never Before), the species captured only in the given year (Only Once), percentage of species represented by only one capture (Only One), Fisher’s alpha (Alpha), and the standard deviation of Fishers alpha (SD).

Year	Days	Captured	Genera	Species	Never before	Only once	Only one	Alpha	SD
2001	133	12,819	219	339	339	19	87(26%)	64.12	1.54
2002	115	6,688	176	257	37	6	68(26%)	53.05	1.56
2003	146	8,094	193	288	36	6	63(22%)	58.29	1.60
2004	121	6,754	175	278	29	13	66(24%)	58.42	1.67
2005	127	6,950	182	274	14	5	39(14%)	56.93	1.63
2006	126	7,067	192	278	11	5	64(23%)	57.73	1.64
2007	164	9,837	199	317	15	8	79(25%)	60.17	1.67
2008	142	7,476	172	263	5	4	60(23%)	53.11	1.52
2009	216	11,892	209	333	15	15	74(22%)	63.59	1.56
All	1290	77,581	2934	501			64(13%)	71.86	1.17

Given that we documented 501 species at this one location, one might suggest that this urban environment had greater macrolepidopteran diversity than 15 of the 19 North American sites in Table 1. However, this comparison is problematic. Sampling effort both within season and the number of seasons affect the number of species collected (Gotelli and Colwell 2001). The other studies used multiple traps in a variety of habitats, but usually did so over a shorter time span both in terms of the number of years sampled and in terms of the number of trapping nights per year. The type of black light trap may also have an influence (Schweitzer et al. 2011; Truxa and Fiedler 2012). Furthermore, many of the sampled habitats in the other studies might be more homogeneous than an urban landscape with corresponding influence on biodiversity (Fox et al. 1997; Krauss et al. 2003; McKinney 2002; Southwood et al. 1979), especially considering that small plantings of native vegetation augment botanical diversity and concomitant increases in moth biodiversity (Burghardt et al. 2008). Considering the large number of species found in this survey, we would agree with the idea that it should be possible for urban planning committees to design urban landscapes that support an abundant and diverse macrolepidopteran fauna (Ockinger et al. 2009; Valtonen et al. 2007), which might also improve the habitat for birds and other wildlife.

There have been three moth surveys near this survey. The Wilderness Center had the fewest number of shared species with our study (Table 5). The greatest similarity was in the Geometridae where 15% of the combined species were in common. The Atwood Lake Park survey and the Funk Bottoms survey were much more like our survey with a 28% or better overlap in species lists (average overlap 52%). If urbanization at the Wooster site had elevated diversity due to habitat fragmentation and colonization by a diverse assemblage of generalists, then one would expect that most of the species unique to the Wooster survey would be in the Noctuidae. However, this was the case only for comparisons with Atwood Lake Park where 46% of the Noctuid moths captured at Wooster were unique to Wooster, while only 29% of the Geometrids were. This is in contrast to Funk Bottoms where 58% of the Noctuids were unique to Wooster while 62% of the Geometrids were unique, and the Wilderness Center had 35% and 38% respectively. This outcome is inconsistent with the hypothesis that urbanization at this location has increased diversity by attracting more generalists at the expense of naturally occurring moths.

**Table 5.** Similarity between our results and those from other surveys in Ohio in numbers of species in each family or subfamily. Arct = Arctiinae, Geo = Geometridae, Noc = Noctuidae, Noto = Notodontinae, Sat = Saturniidae, Sphing = Sphingidae.

Location	Arct	Geo	Noc	Noto	Sat	Sphing
Funk Bottoms						
In Common	14	40	100	18	6	4
Unique to cited	2	6	25	1	1	1
Unique to ours	6	64	161	15	2	9
Wilderness Center						
In Common	16	64	191	28	6	10
Unique to cited	3	32	51	8	0	4
Unique to ours	3	40	117	5	2	3
Atwood Lake Park						
In Common	14	74	135	22	5	9
Unique to cited	1	19	37	5	2	2
Unique to ours	6	30	126	11	3	4

An alternative strategy to assess the value of this urban moth assemblage is to examine specific genera within the Noctuoidea that are not associated with typical urban vegetation. Larvae from moths in the Noctuid genus *Schinia* are mostly associated with plants in the Asteraceae and Fabaceae. Species in the genus *Catocala* are specialists on plants in the Fabaceae, Fagaceae, Rosaceae, Juglandaceae, Myricaceae, and Salicaceae. The genus *Acronicta* larvae feed on woody shrubs and trees, some are specialists. Larvae of moths in the genus *Papaipema* are borers in stems of plants in the Asteraceae and other weedy species. The tribe Psaphidini primarily feed on members of the Juglandaceae and Fagaceae with the exception of *Copivaleria grotei* (Morrison), which feeds on ash. The subfamily Herminiinae is a member of the family Erebidae, with larvae that primarily feed on senescent plant material (http://www.eeb.uconn.edu/people/wagner/USDA Noctuid Guide Most Current.doc). Table 6 compares the number of species within these groups collected at the various locations. There doesn’t appear to be any pattern. For example, in the *Papaipema* there were six species not recovered in this survey but present in one or more of the other surveys: *Papaipema lysamachiae* Bird, *Papaipema rigida* (Grote), *Papaipema rutila* (Guenée), *Papaipema unimoda* (Smith), *Papaipema marginidens* (Guenée), *Papaipema nelita* (Strecker), and *Papaipema birdi* (Dyar). In the case of Turkey Run (E in Table 6) and Coopers Rock (F in Table 6), half of the species in each case were in common with this survey, and only two species were in common between the two cited works. No species of *Papaipema* was common to all locations, though *Papaipema inquaesita* (Grote & Robinson) was only missing from the Funk Bottoms survey (D in Table 6). Finally, the *Papaipema* in this study are moderately abundant with records of 2 to 90 specimens in total over the nine year survey. The agricultural pest *Papaipema nebris* (Guenée) ranked fifth in abundance within this group. The point is that there does not seem to be a pattern that would indicate that the Wooster moth fauna are lacking in species associated with non-urban environments. It is possible that the abundance of these species is lower in Wooster than in more natural settings. However, the other local surveys did not record abundance data.

**Table 6.** Number of species collected from specific groups for several faunal surveys. These groups contain a large proportion of specialists that could be adversely impacted by urbanization.

Source	Schinia	Catocala	Acronicta	Papaipema	Psaphidini	Herminiinae
A	3	26	23	10	2	25
B	1	16	14	12	2	18
C	2	19	19	8	2	11
D	2	11	5	13	2	11
E	2	12	19	6	2	31
F	0	15	20	6	2	25

A) Current Study B) Atwood Lake (Rings and Metzler 1988) C) Wilderness Center (Rings et al. 1987) D) Funk Bottoms (Williams et al. 1977) E) Turkey Run (Steury et al. 2007) F) Coopers Rock (Butler and Kondo 1991).

### Temporal distribution

**Figure 1.**
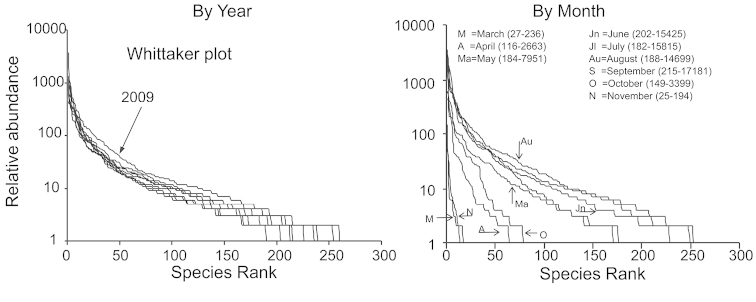
Whittaker plots for both year (where month is ignored), and month (where year is ignored). The number in parentheses is the total number of sample days and total number of captures in that month from 2001 through 2009.

**Figure 2.**
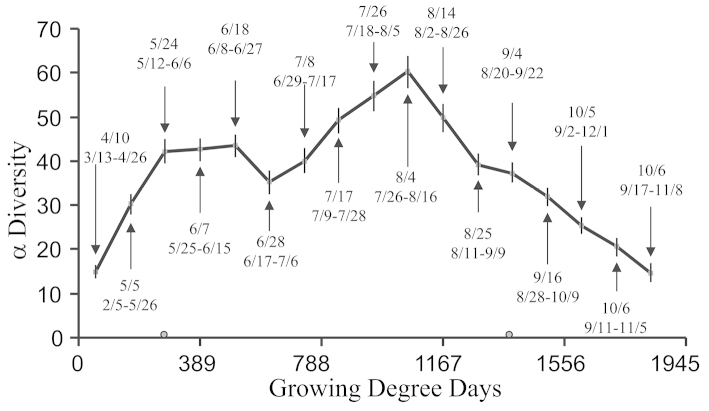
Seasonal and yearly change in a diversity. Bars show the 95% confidence interval. The top date for season was the average date for the midpoint, while the bottom dates give the range in month/day format.

**Figure 3.**
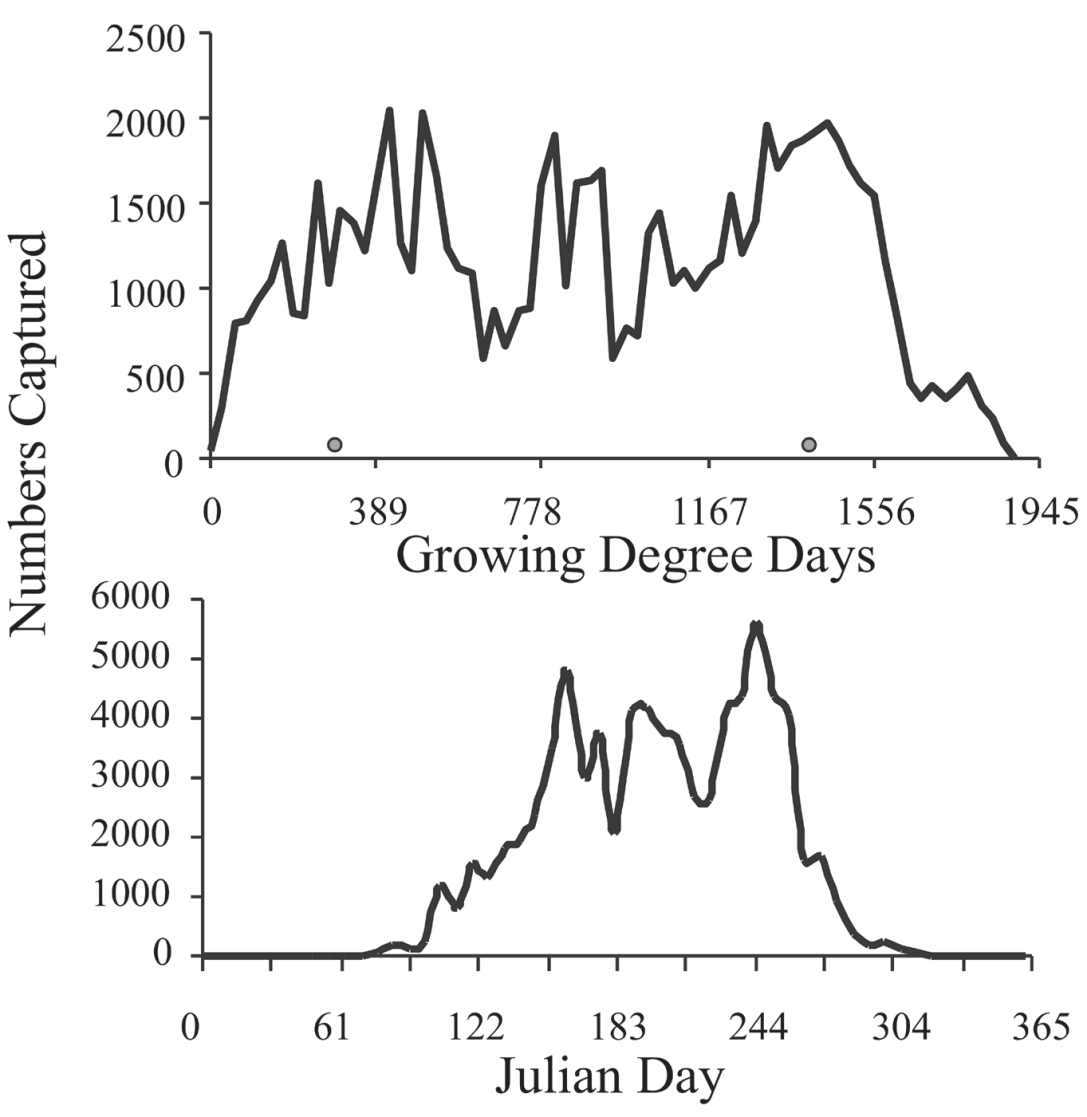
Seasonal abundance by growing degree days and Julian Date.

A Whittaker plot showed no obvious difference in ranked abundance between any of the years (Fig. 1). A Whittaker plot for each month showed that September and October had the most even distribution, and evenness decreased on either side (Fig. 1). Alpha diversity was greatest in early August (Fig. 2). Abundance of all macrolepidoptera had three peaks (Fig. 3). The first and last peaks were caused by the emergence of abundant bivoltine moth species, whereas the central peak was caused by abundant univoltine species. The first peak was between 250 and 583 GDD, the second between 750 and 1111 GDD, and the third between 1334 and 1611 GDD. This corresponds to late June, early August, and late September.

We calculated the number of species that went missing from one year to the next expressed as a proportion of the number of species originally present (e.g., 100* number of species in 2001 not collected in 2002 divided by the total number of species collected in 2001). We also calculated the number of immigrants expressed as a proportion of the number of species present in the year of collection (e.g., 100* number of species in 2002 not collected in 2001 divided by the total number of species in 2002). The missing rate averaged 24.0% (standard deviation 0.0701) while the immigration rate averaged 23.9% (standard deviation 0.0632) from 2001 through 2009. This would suggest that the biodiversity in this area was relatively stable over this nine year period.

**Figure 4.**
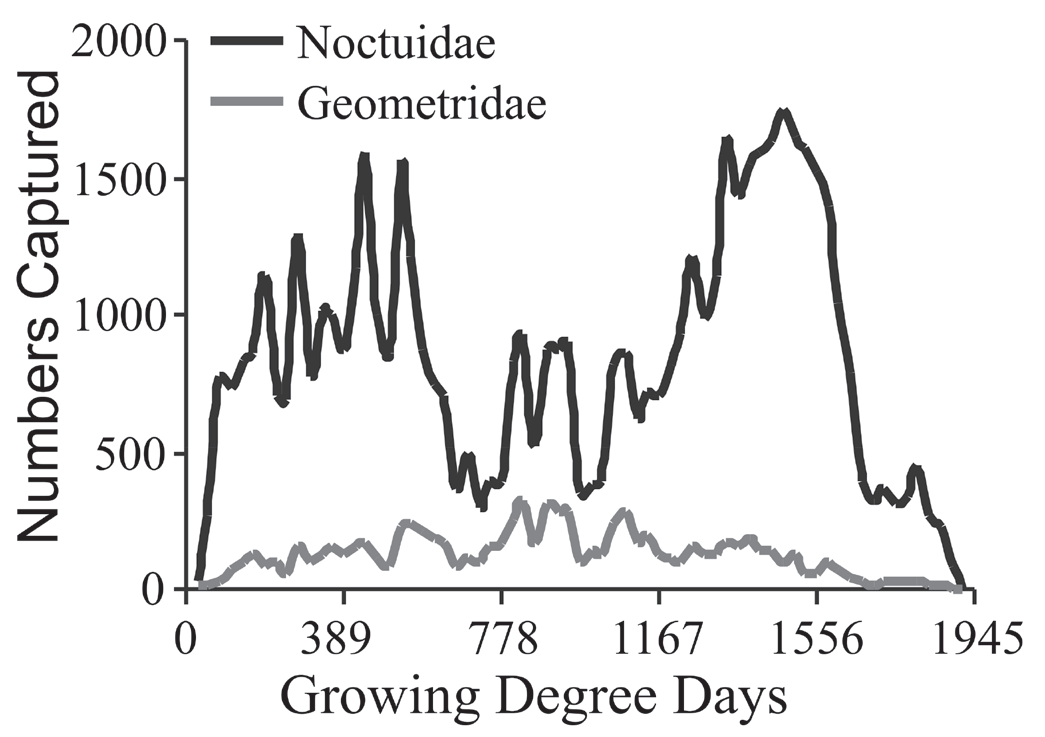
Seasonal abundance based on growing degree days for Noctuidae, and Geometridae.

The Noctuidae had three peak abundances in the year, with the first peak ending at about 722 GDD (late June), a second peak from 722 to 1056 (early August), and the third peak from 1056 GDD onwards (Fig. 4). The first and third peaks were the most substantial, with the third peak containing about 1/3rd more individuals than the first peak. In contrast, Geometrid abundance gradually increased through July, and declined thereafter. The Geometridae lacked the sharp peaks seen in the Noctuidae (Fig. 4).

**Figure 5.**
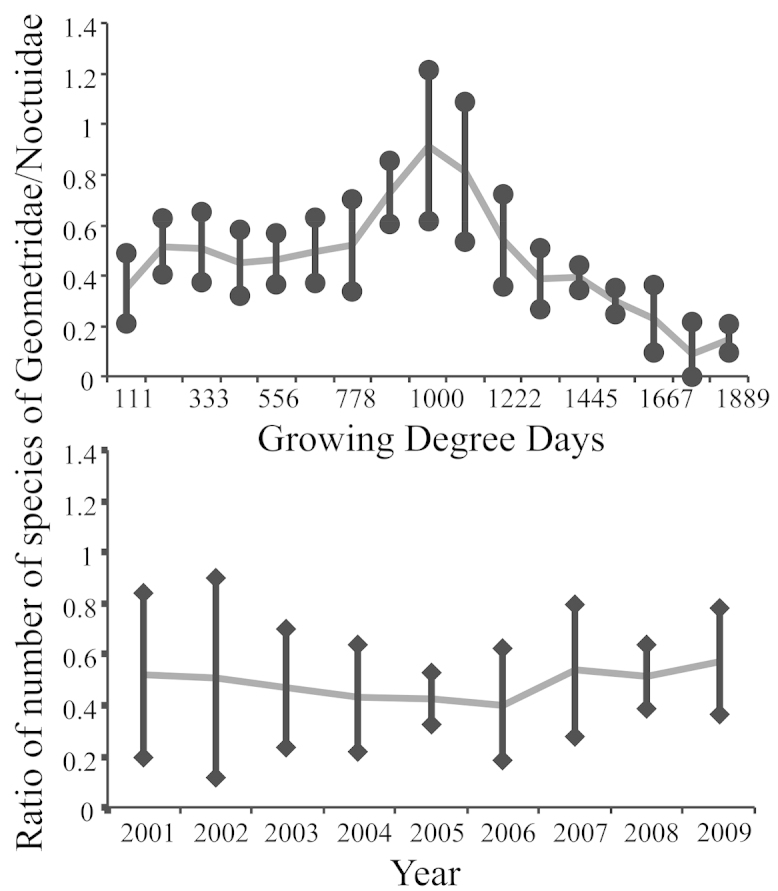
Seasonal and yearly fluctuation in the geometrid/noctuid ratio. Bars are one standard deviation from the mean.

The presence of seasonal patterns has been documented previously, though the specific pattern may be unique to a specific location (Szabo et al. 2007). Because diversity in both Noctuidae and Geometridae had a seasonal component, there was also a seasonal component to the ratio of these two groups (Fig. 5). Although the yearly average was nearly constant, the seasonal ratio ranged from 0.09 to 0.91. Thus a small mismatch in season could result in finding significant differences that are an artifact of seasonality interacting with an experimental design. Seasonal changes in biodiversity could introduce a potentially large inadvertent bias into biodiversity research that was based on a sampling a few dates each year (Summerville 2008). Our data provided a concrete example of this bias. We noted the peak in alpha diversity was in early August (Fig. 2). Yearly alpha diversity (Table 4) was less than peak seasonal alpha diversity (Fig. 2), but yearly alpha diversity would overestimate seasonal diversity through most of a season. For this reason one needs to know where sampling has taken place relative to the seasonal shift in alpha diversity if one is to make valid comparisons with similar studies. Otherwise one does not know if differences between studies represent ecological differences or a mismatch in seasonality. We note in Fig. 2, that if one is sampling at peak alpha diversity, then a shift of only two weeks could result in a significant difference. What might be required to cause such a shift? The current total GDD achieved by August 4 could be achieved by July 25 if every hourly observation was raised by 1.53 °C, or if both minimum and maximum daily temperatures were increased by 1.50 °C. Roughly, this is the equivalent of changing elevation by 153 to 416 m based on an environmental lapse rate of 3.6 to 9.8 °C/1000 m (Sheridan et al. 2010; Varmghani 2012). Alternatively, one could drive about 217 km closer to the equator, assuming a change of 6.9 °C/1000 km (Colwell et al. 2008; Jump et al. 2009). In the current context, this also means that we cannot determine how much of the difference between our results and the three previous surveys was due to the collection dates versus biological differences. Furthermore, the simple approach of first selecting only sampling dates that our study has in common with the other studies will not work to overcome this problem, although it would solve the problem of unequal sampling effort.

A long term trapping effort is managed as the Hungarian Plant Protection and Forestry Light Trap Network (Szentkiralyi 2002; Szontagh 1975). Results from 55+ traps per year sampled from 1962 through 2006 were recently published (Gimesi et al. 2012). They showed three peaks in the number of captured individuals, although in their case the central peak was much larger than the other two. The Hungarian data had a broad peak in Fisher’s alpha corresponding to warmer summer months, and there was a strong relationship between mean daily temperature and biodiversity. This pattern was present in our data (Fig. 2), but in our results alpha had a distinct peak in early August. Seasonal shifts, multiple traps over a broad geographic range, and averaging results over longer time spans would tend to smooth out seasonal trends into a much broader peak in Fisher’s alpha. The Hungarian data showed distinct losses in both abundance and diversity over this period, but one could find nine year spans in their data where abundance and diversity increased (Szentkiralyi 2002). Furthermore, the Hungarian data showed that seasons have gotten earlier by about 2 to 3 weeks over a 44 year span (Gimesi et al. 2012). Based on these results, our inability to detect a similar trend in our data could be due to having only nine years of data.

We were interested in the difference between using a growing degree day model versus a calendar date. We selected 37 individuals with 350 or more captures in the nine year study, and calculated the average day of capture. For each species we divided the mean by the standard deviation, and used a paired t-test for a significant difference between using Julian day versus GDD (df 36; t=7.12; Pr>|t|<0.001). On average there was a 57% reduction in this ratio for GDD relative to using Julian Day (95% CI: 51.7 to 61.3%). Therefore, the GDD approach should significantly increase the statistical power of tests for treatment differences relative to using calendar date.

Looking at the number of *Catocola*, *Acronicta*, and species in the Herminiinae that we collected relative to surveys from less disturbed environments, we would conclude that our sample from an urban environment was not inflated by a large number of generalists attracted to the mix of exotics in the urban landscape. Therefore we would concur with others that urban landscapes can be planned to increase biodiversity relevant to more natural ecosystems (Ockinger et al. 2009; Valtonen et al. 2007). Our survey showed that there were three peaks in moth abundance, whereas biodiversity had a single peak late in the year. We also showed that moth biodiversity was relatively stable with nearly equal missing and immigrating species from year to year. There was also a regular progression of species throughout the year. The sequential gain and loss of species each month resulted in seasonal shifts in the Geometrid:Noctuid ratio such that it is unlikely to be generally useful as a single number describing habitat disturbance. Furthermore, the seasonality demonstrated in these data would suggest that any species ratio would need careful validation prior to use. In describing our data, we made use of a growing degree day (GDD) model. This has the effect of rescaling the data (see Fig. 3). It also corrects for temperature dependent shifts in moth biology. Consequently, some of the variability in the data was removed and this should improve the power of statistical tests involving survey data.

There is no end point to general surveys. No matter how many years of sampling, there will always be an additional species that can be added to the list if sufficient effort is expended. One reason for making such lists is that they provide quantifiable justification for maintaining a natural area to preserve biodiversity. In some cases a threatened local population is being preserved, and those individuals may be locally abundant. More commonly we are preserving rare species associated with a specific habitat. In this case, there is no end to the survey because it is not possible to identify all the species present at an instant in time nor is it possible to identify all the potential species that could live in that habitat. Partly this is a function of forces like climate change, but there are also changes in the spatial distribution of all plant communities. An end point might be reached if the survey goal is to identify those species visitors to the park are likely to encounter and ask “what is this?” In this survey there were 122 species encountered every year. Five species stop flying in May. Five only start flying in August, and three start flying in September. So one could ask how many years it would take to get all 122 species by sampling once per month from May through October. Sampling the first day of these six months will result in recovering an average of 63.2 of these 122 species in any one year. This sampling protocol will only recover 117 of these species in the nine years sampling took place. How does this answer change if we took two or three samples each month? What if we shifted the sampling dates by a few days? Another simple option is to choose the date with the most number of species for the year. In this study that date would fall between 24 July and September 1. The maximum number of species recovered on a single night averaged 51.1. Thus a simple sampling design has difficulty recovering species that we know are present every year. The required sampling effort increases greatly if one desires to go beyond a species list to an understanding of the underlying relationships between these ecologically important organisms.

## Supplementary materials

Supplementary material 1 Nightly moth captures in Wooster, Ohio and summaries of these data Data type: Excel workbook with multiple worksheets Explanation note: The first worksheet on the left is the “Introduction”. The next page to the right in the file includes the weather data. Then there are nine pages with yearly capture data. These pages include the number of growing degree days accumulated by each collection date. Cells are blank if no individuals of a given species were captured on a specific date. Next is a “Condensed List” that contains total number of each species, and the number of years each species was collected. This page contains the species as they were identified and the equivalent under the system by Lafontaine and Schmidt (2010). It also lists the range in capture date, and range in growing degree days. Then follows total captures per year, and a list of known host plants. The next worksheet is a list of the 13 county records and their collection date. Next is a page with a list of univoltine and bivoltine species selected based on abundance and environmental fidelity. Next is a listing of the 20 pest species and their yearly abundance. This was extracted from the main list to facilitate access. Lastly is a page with the moon phases. We did not find the moon phase data of any use, but it may prove useful to someone else. This dataset is made available under the Open Database License (http://opendatacommons.org/licenses/odbl/1.0/). The Open Database License (ODbL) is a license agreement intended to allow users to freely share, modify, and use this Dataset while maintaining this same freedom for others, provided that the original source and author(s) are credited.Roger A. Downer, Timothy A. Ebert
